# Extending Employment beyond the Pensionable Age: A Cohort Study of the Influence of Chronic Diseases, Health Risk Factors, and Working Conditions

**DOI:** 10.1371/journal.pone.0088695

**Published:** 2014-02-19

**Authors:** Marianna Virtanen, Tuula Oksanen, G. David Batty, Leena Ala-Mursula, Paula Salo, Marko Elovainio, Jaana Pentti, Katinka Lybäck, Jussi Vahtera, Mika Kivimäki

**Affiliations:** 1 Centre of Expertise for the Development of Work and Organizations, Finnish Institute of Occupational Health, Helsinki and Turku, Finland; 2 Department of Epidemiology and Public Health, University College London, London, United Kingdom; 3 Centre for Cognitive Ageing and Cognitive Epidemiology, University of Edinburgh, Edinburgh, United Kingdom; 4 Alzheimer Scotland Dementia Research Centre, University of Edinburgh, Edinburgh, United Kingdom; 5 Institute of Health Sciences, University of Oulu, Oulu, Finland; 6 Department of Psychology, University of Turku, Turku, Finland; 7 Service System Department, National Institute for Health and Welfare, Helsinki, Finland; 8 Statistical Services Unit, Keva, Helsinki, Finland; 9 Department of Public Health, University of Turku and Turku University Hospital, Turku, Finland; 10 Institute of Behavioural Sciences, University of Helsinki, Helsinki, Finland; Erasmus University Rotterdam, The Netherlands

## Abstract

**Background:**

In response to the economic consequences of ageing of the population, governments are seeking ways with which people might work into older age. We examined the association of working conditions and health with extended employment (defined as >6 months beyond the pensionable age) in a cohort of older, non-disabled employees who have reached old-age retirement.

**Methods:**

A total of 4,677 Finnish employees who reached their old-age pensionable date between 2005 and 2011 (mean age 59.8 years in 2005, 73% women) had their survey responses before pensionable age linked to national health and pension registers, resulting in a prospective cohort study.

**Results:**

In all, 832 participants (17.8%) extended their employment by more than 6 months beyond the pensionable date. After multivariable adjustment, the following factors were associated with extended employment: absence of diagnosed mental disorder (OR 1.25, 95% confidence interval = 1.01–1.54) and psychological distress (OR 1.68; 1.35–2.08) and of the work characteristics, high work time control (OR 2.31; 1.88–2.84). The projected probability of extended employment was 21.3% (19.5–23.1) among those free of psychiatric morbidity and with high work time control, while the corresponding probability was only 9.2% (7.4–11.4) among those with both psychiatric morbidity and poor work time control. The contribution of chronic somatic diseases was modest.

**Conclusions:**

In the present study, good mental health in combination with the opportunity to control work time seem to be key factors in extended employment into older age. In addition, high work time control might promote work life participation irrespective of employees' somatic disease status.

## Introduction

In most high-income countries, life expectancy is increasing alongside with low birth rates, which means that there are fewer workers in the labour market to cover increasing health care and social security costs [1], [2]. Recently, governments in many countries have started to reform public pension systems in order to extend work careers and avoid ‘the pensions crisis’ [1]. The reforms focus on removing financial incentives for ageing employees to leave the labour force early [3] while various social reasons have been advanced as an incentive to successful ageing, such as the maintenance of social integration and self-esteem [4], [5].

However, little is known about the incentives that would extend the employment beyond the pensionable age (i.e., the age at which people can first draw full benefits without actuarial reduction for early retirement) [1]. In addition to ‘pushing factors’ such as chronic diseases and poor working conditions [6]–[8], ‘pulling factors’ towards retirement exist, such as one's spouse not working, expectations of leisure time, and duties involving care-taking of relatives [8], [9]. Chronic diseases, low socioeconomic status, shift work, job dissatisfaction, and psychosocial stress at work have all been associated with intentions to retire early [10]–[13], early retirement [14]–[19], and work disability pensions [20]–[23]. However, it remains unclear whether a beneficial status in these factors predicts retirement decisions among employees who continue working until the old-age retirement or beyond.

As work disability pensions only account for a minority of all pensions each year, a better understanding is needed of the predictors of extended employment among the remaining working population; that is, those approaching old-age retirement [1]. Even a minor increase in non-disability pensionable age could stabilize the ratio of workers to non-workers [24]. Accordingly, we conducted a large prospective cohort study of a wide range of ‘pushing’ and ‘pulling’ factors possibly associated with the voluntary choice of working longer.

## Methods

### Ethics Statement

The ethics committee of Helsinki and Uusimaa Hospital District approved the study. The response to a questionnaire acts as a form of written informed consent. All data were analysed anonymously.

### Study Context

This study was undertaken in Finland where, at the beginning of 2005, employees received the right to choose age-based retirement between the ages of 63 and 68, with substantial financial advantage for those delaying retirement until after their individual pensionable age. This study was undertaken to assess the retirement choices of employees who were eligible to old-age pension after 1^st^ January, 2005. According to the new pension scheme, the pension accrual rises between the ages 63 and 68, with a more rapid increase after the age of 65. Between the ages of 53 and 62, the standard accrual for old-age pensions is smaller. An individual pensionable retirement date is assigned to all employees, but each employee is free to choose to retire at any time between the age of 63 and 68. The pensionable age can in some cases be lower than 63, depending on the occupation (e.g. fire-fighters) and the employee's job entry year. In cases when the pensionable retirement age is higher, for example, 64 years, employees may still legitimately retire at the age of 63, but pension earnings will be slightly lower. At the age of 60/61, an employee may also choose a voluntary part-time pension which is not disability-based.

### Participants and Procedure

**Figure 1:**
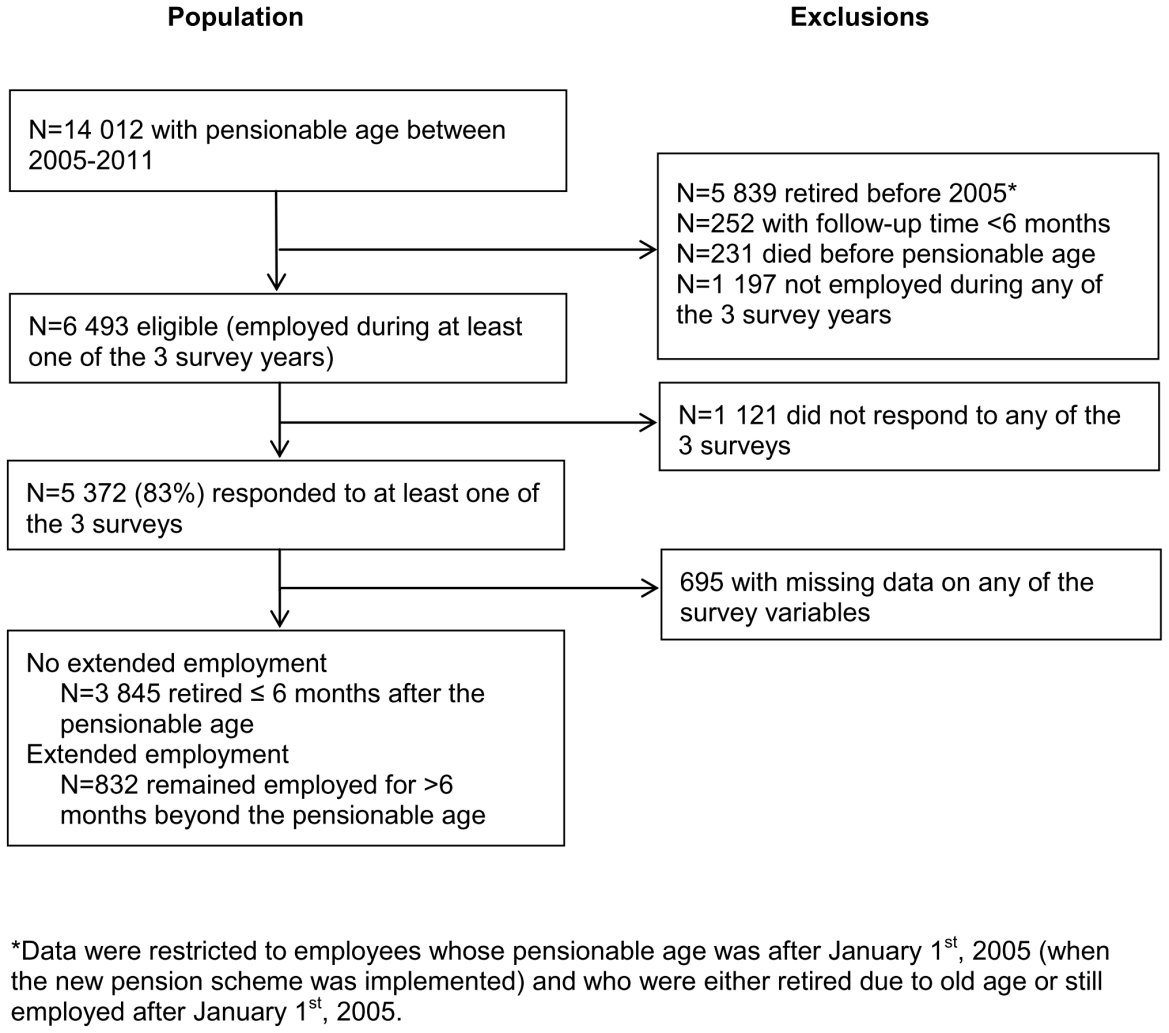
Sample selection procedure.

This study was part of the Finnish Public Sector Study [23], [25], coordinated by the Finnish Institute of Occupational Health. It is a prospective epidemiological cohort study of employees working in 10 towns and 21 hospitals. Employers' records have been used to identify the eligible employees for a cohort; questionnaire surveys have been repeated approximately every 2–4 years, starting from year 2000. We included participants from 10 towns only because work time control was not measured in the surveys of hospital employees. The sample selection procedure is presented in Figure 1. The eligible population comprised 5 372 municipal employees who: 1) were employed for at least six months during any year between 1991 and 2005; 2) were alive and either retired due to old age between 2005 and the end of 2011, or were employed for over six months beyond their individual pensionable age between 2005 and the end of 2011; and 3) during their employment responded to at least one of the study surveys administered in 2000–01, 2004, and 2008 (all employees with at least 6 months' job contract irrespective of their type of job contract were eligible to respond to the survey). Participants who took early retirement through the early retirement scheme and those who were granted either a part time or full time disability pension were excluded. We linked the participants' latest survey response and the employers' records to national pension and health registers. Altogether 4 677 participants (1 286 men, 3 391 women) had complete data on all study variables. Their mean age at the beginning of follow-up was 59.8 (SD = 2.1) years and the mean time between the last survey and the individual pensionable date was 3.7 (SD = 2.6) years. The individual pensionable age was 57–60 years for 9%, 61–63 years for 30%, and 64–65 years for 61% of the participants.

### Measures

We obtained information on the individual pensionable retirement date assigned to each participant from the pension insurance institute for the public sector in Finland (Keva), and on the type and date of actual retirement from the register kept by the Finnish Centre for Pensions, which covers all pensions in Finland. We defined those who stayed employed for over six months beyond the individual pensionable date (from which the pensionable age was derived; ranging from 57 to 65 years) as cases of ‘extended employment’. ‘Not extended’ denote those who retired before six months had passed from the pensionable date.

We examined several ‘pushing’ and ‘pulling’ factors for retirement. Data on chronic diseases were obtained from several national registers: Social Insurance Institution of Finland (prescribed medicines and entitlements to special reimbursements for a chronic disease and diagnosis-specific sickness absence of >9 days), National Institute for Health and Welfare (diagnosis-specific hospitalization), and the Finnish Cancer Registry (cancer). The register-based information covered five years before the individual pensionable date and was successfully linked to all participants. The following major diagnosed chronic diseases were assessed: cardiovascular diseases (coronary artery disease, chronic heart failure, stroke), chronic hypertension, diabetes, mental disorders, musculoskeletal disorders, cancer, and asthma. Data on cardiovascular diseases, diabetes, mental disorders, musculoskeletal disorders, and asthma were completed by survey responses to a check-list of doctor-diagnosed chronic diseases.

Psychological distress was measured using the GHQ-12 (12-item version of the General Health Questionnaire) [26]; the cut-off point for being included as a case was set at ≥4 points [27]. Sleep disturbances were based on the Jenkins Sleep Problem Scale with four different insomnia symptoms (difficulties initiating sleep, waking up several times per night, awakening too early in the morning, and non-refreshing sleep) [28]. We defined disturbed sleep as at least one insomnia symptom at least twice a week during the last month [29], or having records of prescribed sleep medication. Usage of pain medication was derived from the drug prescription register (purchased prescribed pain-killers).

The behaviour related risk factors were current smoking (yes/no), alcohol use above recommended limits (no/yes but ≤192 g (women), 288 g (men) per week/yes and >192 g, 288 g per week) [30], obesity (body mass index of ≥30 kg/m^2^, based on self-reported height and weight), and leisure-time physical activity (<2.0, 2–4.25, >4.25 metabolic equivalent task [MET] hours per day); one MET hour corresponding to approximately 30 min of walking per day) [31].

Socioeconomic status (manual [e.g., cleaners, maintenance workers], lower grade non-manual [e.g., registered nurses, technicians], higher grade non-manual [e.g., teachers, physicians]) and information on age, sex, and type of employment contract (permanent, fixed-term) [32] were derived from the employers' records. Data on taking voluntary, non-disability-based part-time pension before full-time retirement (yes/no) was extracted from the national pension register. Information on work schedule (job without night shifts versus job with night shifts) [33], [34], psychosocial stress factors at work, i.e., job strain (difference between job demands and job control) [35], [36], effort-reward imbalance (ratio between efforts spent and rewards gained) [37], and work time control [23], [38]; mean score of seven items concerning employee's influence over starting and ending times of the work day, breaks during the day, scheduling the shifts and total working hours were all derived from standard questionnaires. We divided the level of job strain, effort-reward imbalance and work time control into tertiles. We obtained the type (rented/owned) and location (metropolitan/non-metropolitan) of residence from the Finnish Population Register Centre and formulated a combined variable (rented, non-metropolitan; rented, metropolitan; owned, non-metropolitan; owned, metropolitan). Marital status (married/co-habiting versus single/widowed/divorced) was obtained from the survey response.

### Statistical Analysis

We analysed morbidity (chronic diseases, symptoms of ill health), health behaviours, working conditions, and socio-demographic characteristics as predictors of extended employment by using logistic regression. To identify robust predictors of extended employment we carried out serial multivariate logistic regression analysis. Finally, to obtain absolute estimates, based on significant predictors in previous analyses, we calculated prediction probability estimates (percentage of participants with extended employment) for four hypothetical cases: 1) poor work and morbidity; 2) poor work without morbidity; 3) good work with morbidity; and 4) good work without morbidity. Because a significant interaction (*P* = 0.046) was found in the main analysis between presence of mental disorder/symptoms and high versus low work time control predicting extended employment, psychiatric morbidity and somatic morbidity in this analysis were examined separately. We derived these estimates from a single logistic regression model that included work characteristics, morbidity and their interaction terms adjusted for all other variables used in the multivariable adjusted models.

## Results

Of the participants, 832 (17.8%) were employed more than 6 months beyond the pensionable date (Table S1 in File S1). The mean age of retirement was 63.1 (SD 1.84) years among those 4436 persons who were already retired at the end of follow-up. Of the 832 persons with extended employment, 591 (71%) had retired and 241 (29%) were still employed at the end of follow-up. Of these 591, the mean extension beyond the pensionable age was 18.4 (SD 6.0–75.6) months (data not shown).

Employees with higher pensionable age, men, those with no spouse, those with a higher socioeconomic status and those living in a rented apartment in the metropolitan area were more likely to extend their employment than others (Table S1 in File S1). Having at least one diagnosed chronic disease was highly common (74.3%) and participants free of any of these conditions had a 1.34-fold (95% CI 1.14–1.59) probability of sustained employment. Absence of any chronic somatic disease was associated with a 1.36-fold (1.16–1.59) probability of extended employment. Diagnosis-specific analyses revealed significant associations with absence of chronic hypertension (1.27; 1.05–1.53), mental disorder (1.26; 1.03–1.53) and musculoskeletal disorder (1.29; 1.11–1.49). Not having symptoms of ill health (psychological distress [1.64; 1.35–2.00], no sleep disturbances [1.22; 1.05–1.42], no pain medication use [1.18; 1.01–1.37]), not having either diagnosed mental disorder or psychological distress symptoms [1.51; 1.28–1.78], and not being obese (1.22; 1.00–1.49) were also associated with extended employment, but no association was found with smoking, alcohol use, or exercise level. Day workers (1.39; 1.01–1.91) and those with no part-time pension (1.51; 1.24–1.83) had greater odds of working longer than their counterparts, as did those who reported low job strain (1.36; 1.12–1.64) and high work time control; the odds ratio of high control over work time compared to low control was 2.37 (1.97–2.87). The non-significant estimates in crude analyses were further adjusted for individual pensionable age, sex, socioeconomic status, marital status and residence and area, but their associations with extended employment were still non-significant (data not shown). Thus, these non-significant predictors were not further examined.

As shown in Table 1, the associations of pensionable age, sex, marital status, socioeconomic status, and residence and area with an extended employment were little affected by multiple adjustments. In terms of health, absence of diagnosed mental disorder (1.25; 1.01–1.54), not having any symptoms of ill health (1.23; 1.02–1.49), absence of psychological distress (1.68; 1.35–2.08), and having neither diagnosed mental disorder nor psychological distress symptoms (1.55; 1.29–1.87) predicted extended employment after all adjustments. Not being on part-time pension (1.65; 1.34–2.03), and regarding psychosocial work characteristics, high work time control (2.31; 1.88–2.84) predicted extended employment.

**Table 1:** Serially adjusted baseline characteristics associated with extended employment at >6 months beyond the pensionable age.

Characteristic	OR*	(95% CI)*	OR†	(95% CI)†	OR‡	(95% CI)‡
Individual pensionable age (years)						
57–60	1.00		1.00		1.00	
61–63	1.73	(1.23–2.45)	1.61	(1.12–2.33)	1.62	(1.12–2.34)
64–65	1.85	(1.32–2.58)	2.00	(1.40–2.87)	2.01	(1.40–2.89)
Sex						
Female	1.00		1.00		1.00	
Male	1.55	(1.30–1.85)	1.41	(1.17–1.69)	1.41	(1.17–1.69)
Marital status						
Married/cohabiting	1.00		1.00		1.00	
Non-married/-cohabitating	1.94	(1.64–2.29)	1.91	(1.61–2.26)	1.92	(1.62–2.28)
Socioeconomic status						
Manual	1.00		1.00		1.00	
Lower grade non-manual	2.03	(1.62–2.54)	1.71	(1.35–2.16)	1.70	(1.34–2.15)
Higher grade non-manual	2.14	(1.71–2.68)	1.77	(1.40–2.23)	1.72	(1.36–2.18)
Residence and area						
Rented, non-metropolitan	1.00		1.00		1.00	
Rented, metropolitan	1.77	(1.33–2.36)	1.71	(1.27–2.28)	1.75	(1.30–2.34)
Owned, non-metropolitan	0.92	(0.69–1.22)	0.93	(0.69–1.23)	0.92	(0.69–1.23)
Owned, metropolitan	1.12	(0.86–1.47)	1.09	(0.83–1.43)	1.09	(0.83–1.44)
Any chronic disease						
Yes	1.00		1.00		1.00	
No	1.30	(1.10–1.54)	1.27	(1.07–1.51)	1.18	(0.99–1.41)
Diagnosed mental disorder						
Yes	1.00		1.00		1.00	
No	1.32	(1.08–1.61)	1.32	(1.08–1.62)	1.25	(1.01–1.54)
Any chronic somatic disease						
Yes	1.00		1.00		1.00	
No	1.30	(1.10–1.53)	1.26	(1.07–1.49)	1.17	(0.99–1.39)
Chronic hypertension						
Yes	1.00		1.00		1.00	
No	1.22	(1.00–1.49)	1.23	(1.01–1.51)	1.15	(0.93–1.41)
Musculoskeletal disorder						
Yes	1.00		1.00		1.00	
No	1.23	(1.06–1.44)	1.20	(1.03–1.40)	1.12	(0.95–1.32)
Any symptoms of ill health						
Yes	1.00		1.00		1.00	
No	1.32	(1.10–1.58)	1.31	(1.09–1.58)	1.23	(1.02–1.49)
Psychological distress						
Yes	1.00		1.00		1.00	
No	1.72	(1.41–2.10)	1.75	(1.42–2.16)	1.68	(1.35–2.08)
Sleep disturbances						
Yes	1.00		1.00		1.00	
No	1.20	(1.03–1.40)	1.19	(1.02–1.40)	1.13	(0.96–1.33)
Pain medication use						
Yes	1.00		1.00		1.00	
No	1.19	(1.02–1.39)	1.19	(1.01–1.39)	1.11	(0.94–1.31)
Diagnosed mental disorder or psychological distress						
Yes	1.00		1.00		1.00	
No	1.59	(1.34–1.88)	1.60	(1.34–1.90)	1.55	(1.29–1.87)
Obesity						
Yes	1.00		1.00		1.00	
No	1.20	(0.98–1.48)	1.26	(1.03–1.56)	1.22	(0.99–1.51)
Work schedule						
Night shifts	1.00		1.00		1.00	
No night shifts	1.05	(0.74–1.50)	0.92	(0.64–1.32)	0.90	(0.62–1.29)
Part-time pension before full retirement						
Yes	1.00		1.00		1.00	
No	1.56	(1.27–1.91)	1.70	(1.38–2.09)	1.65	(1.34–2.03)
Job strain						
High	1.00		1.00		1.00	
Average	1.16	(0.96–1.41)	1.13	(0.92–1.38)	1.11	(0.91–1.36)
Low	1.17	(0.96–1.43)	1.10	(0.89–1.37)	1.07	(0.86–1.33)
Work time control						
Low	1.00		1.00		1.00	
Average	1.31	(1.06–1.61)	1.37	(1.11–1.69)	1.36	(1.10–1.68)
High	2.08	(1.71–2.53)	2.23	(1.82–2.74)	2.31	(1.88–2.84)

OR, odds ratio; CI, confidence interval.

*Adjusted for individual pensionable age, sex, socioeconomic status, marital status, and residence and area.

† As previous+adjusted for type of employment contract, work schedule, part-time pension, job strain, effort-reward imbalance, and work time control.

‡ As previous+adjusted for chronic disease, symptoms of ill health, smoking, alcohol use, leisure time physical activity, and obesity.

**Figure 2:**
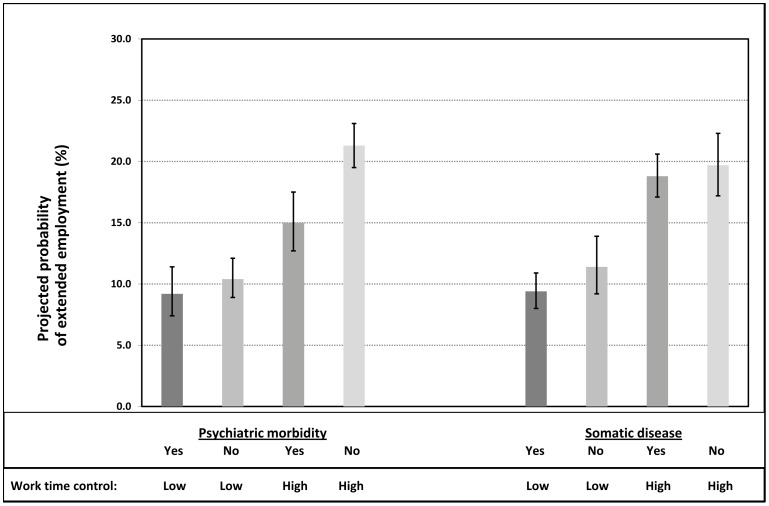
Projected probability (%) and 95% confidence intervals for extended employment at >6 months beyond the pensionable age by psychiatric and somatic morbidity and work time control, multivariable adjusted estimates.

Figure 2 illustrates the projected probability of extended employment according to 1) presence of diagnosed mental disorder or symptoms of psychological distress and work time control and 2) presence of chronic somatic disease and work time control. The projected multivariable-adjusted probability of extended employment was 9.2% (7.4–11.4) for those with psychiatric morbidity and low work time control (the left-hand side of Figure 2). Having low work time control without and having high work time control with psychiatric morbidity were associated with a likelihood of extended employment of 10.4% (8.9–12.1) and 15.0% (12.7–17.5), respectively. The projected probability for those with high work time control and no psychiatric morbidity was 21.3% (19.5–23.1). The corresponding multivariable adjusted odds ratios of extended employment with the reference group of ‘low work time control and psychiatric morbidity’ were for ‘low work time control and no psychiatric morbidity’ 1.16 (0.84–1.61); for ‘high work time control and psychiatric morbidity’ 1.89 (1.35–2.66) and for ‘high work time control and no psychiatric morbidity’ 3.27 (2.41–4.45) (data not shown).

High work time control seemed to benefit employees equally irrespective of their somatic disease status (the right-hand side of Figure 2). The probability of extended employment among employees with high work time control and somatic disease was 18.8% (17.1–20.6); for those with high work time control and without somatic disease it was 19.7% (17.2–22.3). The corresponding multivariable adjusted odds ratios of extended employment with the reference group of ‘low work time control and somatic disease’ were for ‘low work time control and no somatic disease’ 1.28 (0.92–1.77); for ‘high work time control and somatic disease’ 2.62 (2.05–3.35) and for ‘high work time control and no somatic disease’ 2.81 (2.11–3.76) (data not shown).

Similar associations were found in separate analyses among men and women although in general, the probability of extended employment was higher among men: with psychiatric morbidity and low work time control, 11.0% of men, 8.6% of women continued employment beyond the pensionable age; with low work time control and no psychiatric morbidity 12.9% of men, 9.6% of women; with high work time control and psychiatric morbidity 16.5% of men, 14.5% of women; with high work time control and no psychiatric morbidity 23.7% of men, 20.4% of women. Corresponding figures for somatic diseases were: low work time control and somatic disease 11.5% of men, 8.7% of women; low work time control and no somatic disease 13.5% of men, 10.6% of women; high work time control and somatic disease 21.2% of men, 18.0% of women; high work time control and no somatic disease 21.7% of men, 19.0% of women (data not shown).

To examine the robustness of our findings, we carried out the following 5 sensitivity analyses, the results presented in Table S2 in File S1: 1) using a minimum of >12-month extension of employment as the outcome (sample 2); 2) after excluding the 1045 participants who were on voluntary part-time pension before full retirement (sample 3); 3) after excluding the 831 participants whose retirement date in the register was >1 year before the pensionable date (sample 4); 4) and 5) after splitting the data according to participants' pensionable age, 63–65 (sample 5) versus 57–62 years (sample 6). All findings were largely similar to those obtained in the main analysis (sample 1) although the analyses of sample 6 seemed to lack some statistical power (n = 1 095).

Because voluntary part-time pension before full time retirement is believed to enable employees to remain longer in the labour market, we further examined whether the above mentioned rather unexpected finding of voluntary part-time pensioners leaving the workforce earlier was explained by differences in health status. We found that those who had taken voluntary part-time pension more often had a diagnosed chronic disease (socio-demographics adjusted OR 1.44, 95% CI 1.22–1.71) and symptoms of ill health (OR 1.59, 1.29–1.97) compared to those who worked full time (data not shown).

## Discussion

We examined the extent to which chronic diseases, self-reported symptoms, health behaviours, and working conditions are associated with extended employment beyond the pensionable age among older, non-disabled employees who reached old-age retirement. A number of predictors were identified; some of them private in character, such as male sex, being not married or cohabitating, and living in a rented apartment in the metropolitan area. However, we also found several work and health-related factors that can potentially be targeted by interventions in attempts to extend work careers. The strongest of these include good mental health, whether assessed by absence of prior diagnosis of mental disorder or self-reported psychological distress symptoms, and employees' ability to influence work time arrangements. Our projections suggest that employees with good mental health status and high work time control had a 21.3% likelihood of working longer; this probability is more than twice as high as that among employees who had both psychiatric morbidity and poor work time control (9.2%).

Developed countries are now in a situation in which people live longer and remain healthier at older ages than before even though the prevalence of chronic diseases is relatively high [1], [2], the prevalence of diagnosed chronic diseases was high in our study as well (74%). However, in this cohort of employees who reached their statutory retirement age, diagnosed chronic somatic diseases played a minor role; none of the somatic diseases was significantly associated with extended employment in the multivariable adjusted model. High work time control associated with extended employment similarly among those who had a somatic disease (18.8% of those with high work time control continued) and those who did not (19.7% of those with high work time control continued). Thus, our results indicate that high work time control promotes work life extension irrespective of employees' somatic disease status. Psychiatric morbidity, in turn, seemed to reduce the likelihood of extended employment among those with high work time control although the probability was still higher compared to those with low work time control (with or without psychiatric morbidity).

As we targeted a non-disabled population, the role of somatic disease in extending employment beyond the pensionable age was probably smaller compared to an analysis which would additionally have included participants who retired early on health grounds. The most severe somatic diseases occurring during earlier years of employment, such as cancer, may have led to labour market exit before pensionable age [23]. In our study, 8% of the participants who were excluded from the sample due to work disability pension or death had cancer while the corresponding prevalence among the included participants was only 3%. Moreover, we assessed both present (e.g. entitlement to special reimbursement for diabetes medication) and past episodes of diseases (e.g. sickness absence due to back pain). High prevalence of chronic diseases in our study may be partly driven by high prevalence of musculoskeletal disorders (55%); this includes a history of musculoskeletal disorders and the present disorder. For comparison, in a population-based sample of Finnish adults, 77% of men and 83% of women aged 55–64 years reported lifetime prevalence of back pain [39].

Some of the socio-demographic characteristics, such as being a male, being non-married or non-cohabiting, having a high socioeconomic status and living in a rented apartment in the metropolitan area also had an independent association. These findings may reflect that work is an important and enjoyable part of life, but also that for some, financial circumstances may necessitate an extension of working life. The latter interpretation is supported by our finding showing extended employment among participants living in a rented apartment in the metropolitan area, a particularly expensive form of dwelling in Finland.

As expected, we found an association between shift work and extended employment, although this association disappeared in multivariable models including numerous other factors, such as socioeconomic status, health-related factors and control over work time. Our measure of work schedule included information on night work only, which probably did not manage to describe all relevant aspects of shift work. However, there is strong evidence suggesting that shift work, particularly when involving night shifts, has adverse effects on metabolic health [8], [33], [34]. Shift work has also been associated with early retirement [14] and disability pension [20].

Control over work time may improve work-life balance among older workers; increased autonomy in working-time regulation helps to suit family and social commitments, as well as possibilities to optimize commuting hours and to adjust personal working capacity by varying working hours in varying life circumstances [13]. In addition to being associated with lower sickness absence and work disability rates [23], [38], [40], high work time control has predicted lower sickness absence among employees who had long domestic, commuting, and working hours [40]. Furthermore, it may alleviate problems arising from the extended time needed for recovery from stress and fatigue caused by work duties in old age [13].

Perceived work stress (‘job strain’ or ‘effort-reward imbalance’) was not associated with extension of employment in this study; of these, job strain was only associated with the outcome to the degree it was related to socio-demographic factors and health indicators. This is not in agreement with studies on early retirement and disability [14]–[17]. It is possible that among employees who reach old-age retirement, the association between job strain and retirement behaviour is mediated by health problems.

It has been suggested that older employees prefer part-time work to standard full-time work [1], [8], and the part-time pension system in Finland has been developed to help older, non-disabled employees to continue in their employment for longer. However, we found that part-time pension before full-time retirement was generally associated with a lower probability of working for longer. Interestingly, a detailed analysis revealed that the part-time pensioners in our cohort had more health problems than their counterparts who had not taken part-time pension before full retirement. It is possible that due to part-time work, these employees succeeded in working until the pensionable age, thus avoiding disability retirement. Further research is needed to determine whether there are differences between occupations, for example nurses and teachers.

The strengths of this study include its large data and prospective design and the fact that we were able, to our best knowledge, for the first time, to focus on employees who reached their pensionable age with potential to either retire on a statutory basis or extend their employment, rather than those who obliged to early retirement schemes or those who retired due to disability. Sensitivity analyses were carried out to address potential bias and they confirmed the main findings. An important limitation, shared by all observational studies, is that we cannot fully eliminate residual confounding by imprecisely measured or unmeasured factors that are associated with the exposure and retirement behaviour. Interventions improving employees' control over work time are therefore needed in order to determine whether the observed associations are causal.

However, we were able to use a register of each employee's individual pensionable date from which we could reliably calculate whether there was an extension in employment by comparing the date with the actual retirement date. The retirement data were based on national registers, which are trustworthy sources of pensionable dates and different types of retirement and their timing. All participants were covered by National Health Insurance, allowing us to follow all participants' health registers irrespective of their employment status. We completed the register data on chronic diseases with self-reported doctor-diagnosed diseases, but still, some undiagnosed and untreated cases may not have been detected. Although the present sample represents well the Finnish municipal workforce (73% women versus 75% women among old-age retirees in the whole municipal sector) [41], our findings may not be fully generalisable to the whole Finnish working population or countries with other pension systems [7].

In conclusion, these data suggest that good mental health in combination with the opportunity to control work time are key predictors of extended employment beyond the statutory retirement age. Furthermore, high work time control might contribute to longer work careers despite the presence of chronic somatic diseases. Further intervention studies are needed to confirm these novel observational findings. If the observed associations are proven causal, policies to improve work life participation among older employees should include the measures of mental health promotion and the improvement of older employees' potential to influence their work time.

## Supporting Information

File S1 **Supporting tables.** Table S1. Association between baseline characteristics and extended employment at >6 months beyond the pensionable age. Table S2. Multivariable adjusted associations between baseline characteristics and extended employment beyond the pensionable age; the main analysis (Sample 1) and 5 sensitivity analyses (Samples 2 to 6).
(DOC)
