# Supplementary material for: Extending Employment beyond the Pensionable Age: A Cohort Study of the Influence of Chronic Diseases, Health Risk Factors, and Working Conditions
Source: PLoS One. 2014 Feb 19;9(2):e88695. doi: 10.1371/journal.pone.0088695 (PMC3929527; doi:10.1371/journal.pone.0088695)
Supplement: File S1 — Supporting tables. Table S1. Association between baseline characteristics and extended employment at >6 months beyond the pensionable age. Table S2. Multivariable adjusted associations between baseline characteristics and extended employment beyond the pensionable age; the main analysis (Sample 1) and 5 sensitivity analyses (Samples 2 to 6). (DOC) [file pone.0088695.s001.doc]

**Table S1. Association between baseline characteristics and extended employment at >6 months**

beyond the pensionable age

| **Characteristic** | **N of cases*** | | **N of participants** | | **Unadjusted probability, %** | | **OR†** | **(95 % CI)‡** |
| --- | --- | --- | --- | --- | --- | --- | --- | --- |
| All | 832 | | 4 677 | | 17.8 | |  |  |
| Socio-demographic factors |  | |  | |  | |  |  |
| Individual pensionable age (years) |  | |  | |  | |  |  |
| 57-60 | 45 | | 420 | | 10.7 | | 1.00 |  |
| 61-63 | 245 | | 1400 | | 17.5 | | 1.77 | (1.26-2.48) |
| 64-65 | 542 | | 2857 | | 19.0 | | 1.95 | (1.41-2.70) |
| Sex |  | |  | |  | |  |  |
| Female | 579 | | 3 391 | | 17.1 | | 1.00 |  |
| Male | 253 | | 1 286 | | 19.7 | | 1.19 | (1.01-1.40) |
| Marital status |  | |  | |  | |  |  |
| Married/cohabiting | 534 | | 3 478 | | 15.4 | | 1.00 |  |
| Non-married/-cohabitating | 298 | | 1 199 | | 24.9 | | 1.82 | (1.55-2.14) |
| Socioeconomic status |  | |  | |  | |  |  |
| Manual | 129 | | 1 111 | | 11.6 | | 1.00 |  |
| Lower grade non-manual | 359 | | 1 920 | | 18.7 | | 1.75 | (1.41-2.17) |
| Higher grade non-manual | 344 | | 1 646 | | 20.9 | | 2.01 | (1.62-2.50) |
| Residence and area |  | |  | |  | |  |  |
| Rented, non-metropolitan | 82 | | 515 | | 15.9 | | 1.00 |  |
| Rented, metropolitan | 213 | | 838 | | 25.4 | | 1.80 | (1.36-2.39) |
| Owned, non-metropolitan | 199 | | 1 367 | | 14.6 | | 0.90 | (0.68-1.19) |
| Owned, metropolitan | 338 | | 1 957 | | 17.3 | | 1.10 | (0.85-1.43) |
| Diagnosed chronic diseases |  | |  | |  | |  |  |
| Any chronic disease |  | |  | |  | |  |  |
| Yes | 578 | | 3 476 | | 16.6 | | 1.00 |  |
| No | 254 | | 1 201 | | 21.2 | | 1.34 | (1.14-1.59) |
| Any chronic somatic disease |  | |  | |  | |  |  |
| Yes | 538 | | 3278 | | 16.4 | | 1.00 |  |
| No | 294 | | 1399 | | 21.0 | | 1.36 | (1.16-1.59) |
| Cardiovascular disease |  | |  | |  | |  |  |
| Yes | 70 | | 407 | | 17.2 | | 1.00 |  |
| No | 762 | | 4 270 | | 17.9 | | 1.05 | (0.80-1.37) |
| Chronic hypertension |  | |  | |  | |  |  |
| Yes | 151 | | 994 | | 15.2 | | 1.00 |  |
| No | 681 | | 3 683 | | 18.5 | | 1.27 | (1.05-1.53) |
| Diabetes |  | |  | |  | |  |  |
| Yes | 56 | | 352 | | 15.9 | | 1.00 |  |
| No | 776 | | 4 325 | | 17.9 | | 1.16 | (0.86-1.55) |
| Mental disorder |  | |  | |  | |  |  |
| Yes | 144 | | 944 | | 15.3 | | 1.00 |  |
| No | 688 | | 3 733 | | 18.4 | | 1.26 | (1.03-1.53) |
| Musculoskeletal disorder |  | |  | |  | |  |  |
| Yes | 413 | | 2 562 | | 16.1 | | 1.00 |  |
| No | 419 | | 2 115 | | 19.8 | | 1.29 | (1.11-1.49) |
| Cancer |  | |  | |  | |  |  |
| Yes | 19 | | 151 | | 12.6 | | 1.00 |  |
| No | 813 | | 4 526 | | 18.0 | | 1.52 | (0.93-2.48) |
| Asthma |  | |  | |  | |  |  |
| Yes | 48 | | 323 | | 14.9 | | 1.00 |  |
| No | 784 | | 4 354 | | 18.0 | | 1.26 | (0.92-1.73) |
| Symptoms of ill health |  | |  | |  | |  |  |
| Any symptoms |  | |  | |  | |  |  |
| Yes | 614 | | 3 646 | | 16.8 | | 1.00 |  |
| No | 218 | | 1 031 | | 21.1 | | 1.32 | (1.11-1.57) |
| Psychological distress |  | |  | |  | |  |  |
| Yes | 140 | | 1 099 | | 12.7 | | 1.00 |  |
| No | 692 | | 3 578 | | 19.3 | | 1.64 | (1.35-2.00) |
| Sleep disturbances |  | |  | |  | |  |  |
| Yes | 453 | | 2 736 | | 16.6 | | 1.00 |  |
| No | 379 | | 1 941 | | 19.5 | | 1.22 | (1.05-1.42) |
| Pain medication use |  | |  | |  | |  |  |
| Yes | 380 | | 2 291 | | 16.6 | | 1.00 |  |
| No | 452 | | 2 386 | | 18.9 | | 1.18 | (1.01-1.37) |
| Diagnosed mental disorder or psychological distress |  | |  | |  | |  |  |
| Yes | 233 | | 1 656 | | 14.1 | | 1.00 |  |
| No | 599 | | 3 021 | | 19.8 | | 1.51 | (1.28-1.78) |
| Behaviour-related risks | |  | |  | |  |  |  |
| Smoking | |  | |  | |  |  |  |
| Yes | | 101 | | 597 | | 16.9 | 1.00 |  |
| No | | 731 | | 4 080 | | 17.9 | 1.07 | (0.85-1.35) |
| Alcohol use | |  | |  | |  |  |  |
| Above recommended limits | | 83 | | 432 | | 19.2 | 1.00 |  |
| Within recommended limits | | 614 | | 3 504 | | 17.5 | 0.89 | (0.69-1.15) |
| No | | 135 | | 741 | | 18.2 | 0.94 | (0.69-1.27) |
| Obesity | |  | |  | |  |  |  |
| Yes | | 137 | | 884 | | 15.5 | 1.00 |  |
| No | | 695 | | 3 793 | | 18.3 | 1.22 | (1.00-1.49) |
| Leisure-time physical activity | |  | |  | |  |  |  |
| Low | | 264 | | 1 506 | | 17.5 | 1.00 |  |
| Average | | 282 | | 1 676 | | 16.8 | 0.95 | (0.79-1.14) |
| High | | 286 | | 1 495 | | 19.1 | 1.11 | (0.92-1.34) |
| Work conditions | |  | |  | |  |  |  |
| Type of employment contract | |  | |  | |  |  |  |
| Fixed-term | | 18 | | 139 | | 13.0 | 1.00 |  |
| Permanent | | 814 | | 4 538 | | 17.9 | 1.47 | (0.89-2.42) |
| Work schedule | |  | |  | |  |  |  |
| Night shifts | | 46 | | 335 | | 13.7 | 1.00 |  |
| No night shifts | | 786 | | 4 342 | | 18.1 | 1.39 | (1.01-1.91) |
| Part-time pension before full retirement | |  | |  | |  |  |  |
| Yes | | 141 | | 1 045 | | 13.5 | 1.00 |  |
| No | | 691 | | 3 632 | | 19.0 | 1.51 | (1.24-1.83) |
| Job strain | |  | |  | |  |  |  |
| High | | 232 | | 1 521 | | 15.3 | 1.00 |  |
| Average | | 302 | | 1 639 | | 18.4 | 1.26 | (1.04-1.51) |
| Low | | 298 | | 1 517 | | 19.6 | 1.36 | (1.12-1.64) |
| Effort-reward imbalance at work | |  | |  | |  |  |  |
| High | | 298 | | 1 637 | | 18.2 | 1.00 |  |
| Average | | 250 | | 1 509 | | 16.6 | 0.89 | (0.74-1.07) |
| Low | | 284 | | 1 531 | | 18.6 | 1.02 | (0.85-1.23) |
| Work time control | |  | |  | |  |  |  |
| Low | | 195 | | 1 575 | | 12.4 | 1.00 |  |
| Average | | 238 | | 1 514 | | 15.7 | 1.32 | (1.08-1.62) |
| High | | 399 | | 1 588 | | 25.1 | 2.37 | (1.97-2.87) |

OR, odds ratio; CI, confidence interval.

*Number (%) of participants who extended their employment beyond the pensionable age.

†Unadjusted odds ratios for extended employment.

‡Unadjusted confidence intervals for extended employment.

**Table S2. Multivariable adjusted associations between baseline characteristics and extended employment beyond the pensionable age; the main analysis (Sample 1) and 5 sensitivity analyses (Samples 2 to 6)**

|  | **Sample 1 (n=4 677)*** | |  | **Sample 2 (n=4 602)†** | |  | **Sample 3 (n=3 632)‡** | |  | **Sample 4 (n=3 846)§** | | **Sample 5 (n=3 582)¶** | |  | **Sample 6 (n=1 095)**** | |
| --- | --- | --- | --- | --- | --- | --- | --- | --- | --- | --- | --- | --- | --- | --- | --- | --- |
| **Characteristic** | **OR** | **(95% CI)** |  | **OR** | **(95% CI)** |  | **OR** | **(95% CI)** |  | **OR** | **(95% CI)** | **OR** | **(95% CI)** |  | **OR** | **(95% CI)** |
| Individual pensionable age (y) |  |  |  |  |  |  |  |  |  |  |  |  |  |  |  |  |
| 57-60 | 1.00 |  |  | 1.00 |  |  | 1.00 |  |  | 1.00 |  | - |  |  | - |  |
| 61-63 | 1.62 | (1.12-2.34) |  | 1.27 | (0.83-1.92) |  | 1.64 | (1.13-2.39) |  | 1.62 | (1.12-2.33) | - |  |  | - |  |
| 64-65 | 2.01 | (1.40-2.89) |  | 1.30 | (0.86-1.96) |  | 2.09 | (1.45-3.01) |  | 2.87 | (2.00-4.12) | - | - |  | - | - |
| Sex |  |  |  |  |  |  |  |  |  |  |  |  |  |  |  |  |
| Female | 1.00 |  |  | 1.00 |  |  | 1.00 |  |  | 1.00 |  | 1.00 |  |  | 1.00 |  |
| Male | 1.41 | (1.17-1.69) |  | 1.25 | (0.99-1.57) |  | 1.37 | (1.11-1.69) |  | 1.34 | (1.11-1.63) | 1.44 | (1.18-1.76) |  | 0.70 | (0.36-1.36) |
| Marital status |  |  |  |  |  |  |  |  |  |  |  |  |  |  |  |  |
| Married/cohabiting | 1.00 |  |  | 1.00 |  |  | 1.00 |  |  | 1.00 |  | 1.00 |  |  | 1.00 |  |
| Non-married/-cohabitating | 1.92 | (1.62-2.28) |  | 2.10 | (1.71-2.59) |  | 1.91 | (1.58-2.30) |  | 1.80 | (1.51-2.15) | 2.01 | (1.66-2.43) |  | 1.50 | (1.00-2.27) |
| Socioeconomic status |  |  |  |  |  |  |  |  |  |  |  |  |  |  |  |  |
| Manual | 1.00 |  |  | 1.00 |  |  | 1.00 |  |  | 1.00 |  | 1.00 |  |  | 1.00 |  |
| Lower grade non-manual | 1.70 | (1.34-2.15) |  | 1.45 | (1.08-1.94) |  | 1.62 | (1.25-2.10) |  | 1.76 | (1.39-2.24) | 1.72 | (1.33-2.22) |  | 1.10 | (0.57-2.16) |
| Higher grade non-manual | 1.72 | (1.36-2.18) |  | 1.73 | (1.29-2.32) |  | 1.70 | (1.31-2.21) |  | 2.39 | (1.86-3.06) | 1.64 | (1.27-2.12) |  | 1.51 | (0.71-3.19) |
| Residence and area |  |  |  |  |  |  |  |  |  |  |  |  |  |  |  |  |
| Rented, non-metropolitan | 1.00 |  |  | 1.00 |  |  | 1.00 |  |  | 1.00 |  | 1.00 |  |  | 1.00 |  |
| Rented, metropolitan | 1.75 | (1.30-2.34) |  | 1.77 | (1.24-2.52) |  | 1.85 | (1.34-2.56) |  | 1.74 | (1.29-2.35) | 1.58 | (1.14-2.18) |  | 2.48 | (1.15-5.35) |
| Owned, non-metropolitan | 0.92 | (0.69-1.23) |  | 0.90 | (0.63-1.28) |  | 0.94 | (0.68-1.29) |  | 0.95 | (0.71-1.29) | 0.81 | (0.59-1.12) |  | 1.44 | (0.71-2.92) |
| Owned, metropolitan | 1.09 | (0.83-1.44) |  | 0.93 | (0.66-1.31) |  | 1.13 | (0.83-1.53) |  | 1.11 | (0.84-1.47) | 0.99 | (0.73-1.34) |  | 1.49 | (0.73-3.01) |
| Any chronic disease |  |  |  |  |  |  |  |  |  |  |  |  |  |  |  |  |
| Yes | 1.00 |  |  | 1.00 |  |  | 1.00 |  |  | 1.00 |  | 1.00 |  |  | 1.00 |  |
| No | 1.18 | (0.99-1.41) |  | 1.17 | (0.94-1.46) |  | 1.15 | (0.94-1.40) |  | 1.18 | (0.98-1.42) | 1.21 | (0.99-1.47) |  | 1.16 | (0.75-1.82) |
| Diagnosed mental disorder |  |  |  |  |  |  |  |  |  |  |  |  |  |  |  |  |
| Yes | 1.00 |  |  | 1.00 |  |  | 1.00 |  |  | 1.00 |  | 1.00 |  |  | 1.00 |  |
| No | 1.25 | (1.01-1.54) |  | 1.29 | (0.98-1.69) |  | 1.20 | (0.95-1.53) |  | 1.23 | (0.99-1.53) | 1.36 | (1.07-1.73) |  | 0.84 | (0.52-1.35) |
| Any chronic somatic disease |  |  |  |  |  |  |  |  |  |  |  |  |  |  |  |  |
| Yes | 1.00 |  |  | 1.00 |  |  | 1.00 |  |  | 1.00 |  | 1.00 |  |  | 1.00 |  |
| No | 1.17 | (0.99-1.39) |  | 1.11 | (0.90-1.37) |  | 1.13 | (0.94-1.37) |  | 1.18 | (0.99-1.41) | 1.19 | (0.99-1.44) |  | 1.17 | (0.76-1.78) |
| Chronic hypertension |  |  |  |  |  |  |  |  |  |  |  |  |  |  |  |  |
| Yes | 1.00 |  |  | 1.00 |  |  | 1.00 |  |  | 1.00 |  | 1.00 |  |  | 1.00 |  |
| No | 1.15 | (0.93-1.41) |  | 1.27 | (9.97-1.65) |  | 1.07 | (0.85-1.35) |  | 1.14 | (0.92-1.41) | 1.07 | (0.85-1.34) |  | 1.49 | (0.84-2.63) |
| Musculoskeletal disorder |  |  |  |  |  |  |  |  |  |  |  |  |  |  |  |  |
| Yes | 1.00 |  |  | 1.00 |  |  | 1.00 |  |  | 1.00 |  | 1.00 |  |  | 1.00 |  |
| No | 1.12 | (0.95-1.32) |  | 1.09 | (0.89-1.33) |  | 1.16 | (0.97-1.39) |  | 1.17 | (0.98-1.38) | 1.13 | (0.95-1.36) |  | 1.12 | (0.75-1.67) |
| Any symptoms of ill health |  |  |  |  |  |  |  |  |  |  |  |  |  |  |  |  |
| Yes | 1.00 |  |  | 1.00 |  |  | 1.00 |  |  | 1.00 |  | 1.00 |  |  | 1.00 |  |
| No | 1.23 | (1.02-1.49) |  | 1.29 | (1.02-1.62) |  | 1.28 | (1.04-1.58) |  | 1.20 | (0.99-1.47) | 1.31 | (1.06-1.62) |  | 0.91 | (0.57-1.47) |
| Psychological distress |  |  |  |  |  |  |  |  |  |  |  |  |  |  |  |  |
| Yes | 1.00 |  |  | 1.00 |  |  | 1.00 |  |  | 1.00 |  | 1.00 |  |  | 1.00 |  |
| No | 1.68 | (1.35-2.08) |  | 1.86 | (1.40-2.46) |  | 1.80 | (1.41-2.30) |  | 1.69 | (1.35-2.11) | 1.69 | (1.33-2.14) |  | 1.70 | (0.99-2.91) |
| Sleep disturbances |  |  |  |  |  |  |  |  |  |  |  |  |  |  |  |  |
| Yes | 1.00 |  |  | 1.00 |  |  | 1.00 |  |  | 1.00 |  | 1.00 |  |  | 1.00 |  |
| No | 1.13 | (0.96-1.33) |  | 1.22 | (1.00-1.50) |  | 1.23 | (1.03-1.47) |  | 1.12 | (0.94-1.32) | 1.12 | (0.94-1.34) |  | 1.20 | (0.81-1.79) |
| Pain medication use |  |  |  |  |  |  |  |  |  |  |  |  |  |  |  |  |
| Yes | 1.00 |  |  | 1.00 |  |  | 1.00 |  |  | 1.00 |  | 1.00 |  |  | 1.00 |  |
| No | 1.11 | (0.94-1.31) |  | 1.25 | (1.02-1.54) |  | 1.14 | (0.95-1.37) |  | 1.09 | (0.92-1.30) | 1.20 | (1.00-1.44) |  | 0.81 | (0.54-1.21) |
| Diagnosed mental disorder or psychological distress |  |  |  |  |  |  |  |  |  |  |  |  |  |  |  |  |
| Yes | 1.00 |  |  | 1.00 |  |  | 1.00 |  |  | 1.00 |  | 1.00 |  |  | 1.00 |  |
| No | 1.55 | (1.29-1.87) |  | 1.62 | (1.28-2.05) |  | 1.58 | (1.28-1.94) |  | 1.54 | (1.27-1.87) | 1.60 | (1.30-1.97) |  | 1.39 | (0.89-2.19) |
| Obesity |  |  |  |  |  |  |  |  |  |  |  |  |  |  |  |  |
| Yes | 1.00 |  |  | 1.00 |  |  | 1.00 |  |  | 1.00 |  | 1.00 |  |  | 1.00 |  |
| No | 1.22 | (0.99-1.51) |  | 1.23 | (0.95-1.59) |  | 1.38 | (1.08-1.77) |  | 1.25 | (1.01-1.56) | 1.15 | (0.92-1.46) |  | 1.89 | (1.03-3.47) |
| Work schedule |  |  |  |  |  |  |  |  |  |  |  |  |  |  |  |  |
| Night shifts | 1.00 |  |  | 1.00 |  |  | 1.00 |  |  | 1.00 |  | 1.00 |  |  | 1.00 |  |
| No night shifts | 0.90 | (0.62-1.29) |  | 0.99 | (0.63-1.53) |  | 0.83 | (0.57-1.21) |  | 0.95 | (0.66-1.37) | 0.97 | (0.57-1.66) |  | 0.65 | (0.39-1.10) |
| Part-time pension before full retirement |  |  |  |  |  |  |  |  |  |  |  |  |  |  |  |  |
| Yes | 1.00 |  |  | 1.00 |  |  | - |  |  | 1.00 |  | 1.00 |  |  | - |  |
| No | 1.65 | (1.34-2.03) |  | 2.34 | (1.74-3.13) |  | - | - |  | 1.77 | (1.43-2.19) | 1.78 | (1.44-2.19) |  | - | - |
| Job strain |  |  |  |  |  |  |  |  |  |  |  |  |  |  |  |  |
| High | 1.00 |  |  | 1.00 |  |  | 1.00 |  |  | 1.00 |  | 1.00 |  |  | 1.00 |  |
| Average | 1.11 | (0.91-1.36) |  | 0.95 | (0.74-1.22) |  | 1.02 | (0.82-1.27) |  | 1.17 | (0.95-1.43) | 1.10 | (0.88-1.37) |  | 1.22 | (0.76-1.96) |
| Low | 1.07 | (0.86-1.33) |  | 1.06 | (0.81-1.38) |  | 0.91 | (0.72-1.16) |  | 1.16 | (0.93-1.46) | 1.04 | (0.82-1.33) |  | 1.42 | (0.84-2.38) |
| Work time control |  |  |  |  |  |  |  |  |  |  |  |  |  |  |  |  |
| Low | 1.00 |  |  | 1.00 |  |  | 1.00 |  |  | 1.00 |  | 1.00 |  |  | 1.00 |  |
| Average | 1.36 | (1.10-1.68) |  | 1.32 | (1.01-1.71) |  | 1.43 | (1.14-1.81) |  | 1.05 | (0.84-1.31) | 1.39 | (1.10-1.77) |  | 1.20 | (0.74-1.96) |
| High | 2.31 | (1.88-2.84) |  | 1.95 | (1.51-2.51) |  | 2.48 | (1.98-3.11) |  | 1.55 | (1.25-1.94) | 2.40 | (1.92-3.01) |  | 1.80 | (1.05-3.07) |

*Sample 1: The main study sample with >6 months’ extended employment beyond the pensionable age as the outcome (n of cases 832), as model 3 in Table 1.

†Sample 2: A subsample with >12 months’ extended employment beyond the pensionable age as the outcome (n of cases 489).

‡Sample 3: A subsample from which part-time pensioners are excluded; >6 months’ extended employment beyond the pensionable age as the outcome (n of cases 691).

§Sample 4: A subsample from which employees with the retirement date >1 year before the pensionable date are excluded; >6 months’ extended employment beyond the pensionable age as the outcome (n of cases 832).

¶Sample 5: A subsample including employees with personal pensionable age of 63-65 years; >6 months’ extended employment beyond the pensionable age as the outcome (n of cases 702).

**Sample 6: A subsample including employees with personal pensionable age of 57-62 years; >6 months’ extended employment beyond the pensionable age as the outcome (n of cases 130).
